# Short-Term Changes in Aroma-Related Volatiles in Meat Model: Effect of Fat and *D. hansenii* Inoculation

**DOI:** 10.3390/foods12122429

**Published:** 2023-06-20

**Authors:** Lei Li, Carmela Belloch, Mónica Flores

**Affiliations:** Institute of Agrochemistry and Food Technology (IATA-CSIC), Agustín Escardino Avenue 7, 46980 Paterna, Valencia, Spain; leili@iata.csic.es (L.L.); belloch@iata.csic.es (C.B.)

**Keywords:** amino acids, yeast, aroma, biotransformation, *Debaryomyces hansenii*

## Abstract

This study assessed the effect of replacing pork lard with coconut oil and *Debaryomyces hansenii* inoculation on the biotransformation of amino acids into volatile compounds in a meat model system. Yeast counts, solid-phase microextraction, and gas chromatography/mass spectrometry were used to assess yeast growth and volatile production, respectively. Yeast growth was confirmed until 28 d, although the volatile profile changed until 39 d. Forty-three volatiles were quantified, and their odor activity values (OAVs) were calculated. The presence of fat and yeasts contributed to differences in volatiles. In pork lard models, a delayed formation of lipid-derived aldehyde compounds was observed, whereas in coconut oil models, the generation of acid compounds and their respective esters was enhanced. Yeast activity affected amino acid degradation, which produced an increase in branched-chain aldehydes and alcohols. The aroma profile in the coconut models was influenced by hexanal, acid compounds, and their respective esters, whereas in pork lard models, aroma was affected by methional (musty, potato) and 3-methylbutanal (green, cocoa). The yeast inoculation contributed to the generation of 3-methylbutanoic acid (cheesy) and phenylethyl alcohol (floral). The type of fat and yeast inoculation produced a differential effect on the aroma.

## 1. Introduction

Amino acids are important precursors for the production of the characteristic flavor in dry-cured meat products. Manufacturing practices submit these products to mild temperature during dry-curing and key aroma compounds can be produced from different reactions. Some aroma compounds are generated through Maillard reactions, as detected in model systems containing free amino acids and glucose that mimic the conditions throughout the dry curing process [1]. The catabolism of amino acids produced by microorganisms in dry-cured fermented meats is also responsible for the production of aroma compounds. *Debaryomyces hansenii*, found at all stages of Spanish fermented sausage manufacture, can produce sulfur compounds (such as dimethyl disulfide, dimethyl trisulfide, and methional) through biochemical reactions from sulfur amino acids [2]. Furthermore, Strecker aldehydes can be produced when reactive carbonyls derived from lipid oxidation reactions react with amino acids [3]. The combined action of phenylalanine with carbonyl and free radicals derived from lipid oxidation also generate benzaldehyde [4]; therefore, the formation of free radicals from different compositions of fatty acids also affects the formation of flavor compounds derived from amino acids.

Among the starter cultures in fermented sausages, *D. hansenii* and *Y. lipolytica* have been used for aroma production with different bacteria [5,6]. Furthermore, the antioxidant effect of *D. hansenii* can affect flavor generation [7]. This effect has been observed in the reduction of oxidation values (thiobarbituric acid reactive substances, TBARS) in fermented sausages [7]; however, there is no information on the formation of carbonyl compounds from fats with different fatty acid compositions nor on their effect on the formation of flavor compounds derived from amino acids in the presence of *D. hansenii* strains.

Using model systems can simulate the conditions of processing by mimicking the complex matrix found in meat products, where proteins and lipids make up most of their composition. Myofibrillar proteins can bind to key aroma compounds in dry-cured meat (3-methyl butanal, 2-methyl butanal, hexanal, and methional) that affect aroma perception [8]. Proteins are not the only molecules affecting the binding process. The fat of products, like dry-fermented sausages (approximately 30% fat content), also affect the formation and release by acting as solvents for generated compounds [9].

Understanding the reactions involved and the production of key aroma compounds in dry-cured meat products, such as fermented sausages, requires considering both the Maillard reactions and lipid oxidation in complex systems, as well as the role of starters in their formation. Accordingly, the objective of this study was to reveal the biochemical mechanisms involved in the production of key aroma compounds in a meat model system that is similar in composition to a dry-fermented sausage, containing myofibrillar proteins, free amino acids, and fat from different origins, as well as being inoculated with a starter culture of *D. hansenii*.

## 2. Materials and Methods

### 2.1. Reagents and Standards

All volatile compounds used for identification and quantitation were purchased from Merck (Darmstadt, Germany).

### 2.2. Preparation of the Yeast Starter

The *D. hansenii* strain L1 isolated from fermented sausage [2] was selected for the study. The yeast were grown in glucose peptone yeast (GPY) medium (glucose 2%, yeast extract 0.5%, peptone 0.5% (Pronadisa, Madrid, Spain)) at 25 °C overnight. Cells were collected using a saline solution (0.9% salt), and the cell suspensions were adjusted using a V-1200 spectrophotometer (VWR, Radnor, PA, USA) at 600 nm to reach a concentration of 10^6^ CFU mL^−1^ in the model systems.

### 2.3. Preparation of the Meat Model Systems

A meat model system similar in composition to a dry-fermented sausage containing myofibrillar proteins, free amino acids, and fat from different origins was prepared. Myofibrillar proteins were obtained from fresh minced meat using the *Longissimus dorsi* muscle without apparent fat or connective tissue. Myofibrillar proteins were isolated from meat and homogenized with 0.1 M of Tris-HCl and 20 mM of ethylenediaminetetraacetic acid (EDTA) at pH 7.0 (1:2 *w*/*v*) using a Krups 577 mixer (Solingen, Germany). The homogenate was centrifuged for 30 min at 10,000× *g* at 4 °C, and the supernatant was discarded. This process was repeated three times to remove the supernatant containing sarcoplasmic proteins. Then, the pellet containing myofibrillar proteins was stored at −20 °C until use for preparation of the meat models. The meat models were prepared with a similar composition in terms of additives and free amino acids, as in fermented sausages [9,10] mixed with the extracted myofibrillar proteins, and different fat types (pork lard (El Pozo, Murcia, Spain) or coconut oil (La Masía, Sevilla, Spain)).

Five meat model systems containing myofibrillar proteins (35%) and fat (15%) were prepared: control (C), pork lard (PL), pork lard with yeast (PLY), coconut oil (VO), and coconut oil with yeast (VOY) (Table 1). The meat model systems were prepared by homogenizing the extracted myofibrillar proteins with a solution adjusted to pH 5 and a_w_ 0.895 containing additives, sodium chloride, nitrate, glucose (Table 1) and free amino acids (Table S1) that were previously sterilized by a vacuum-driven filtration system (0.22 μm, 500 mL, cellulose filter) (Grynia, Labbox Labware, Barcelona, Spain) using a Krups 577 mixer (Solingen, Germany). Then, pork lard or coconut oil was added and mixed until an emulsion was obtained. The PLY and VOY models were inoculated with *D. hansenii* L1 (10^6^ CFU ml^−1^). Each meat model system (255 g) was prepared and distributed in sterile Erlenmeyer flasks under sterile conditions (MSC-Advantage, Thermo-Fisher, Waltham, MA, USA). The model systems were incubated at 30 °C, and the experiment was performed in three independent replicates. Samples of 35 g were taken for analyses at 0, 7, 14, 28, and 39 d from each model and replicated. Five grams of sample were used for microbial analysis, 30 g were centrifuged at 16,000 rpm for 30 min at 4 °C to remove protein, fat, and yeast cells, and the supernatant was used first for pH measurement (pH meter 50 VioLab, LabProcess, Barcelona, Spain) and then stored at −20 °C for volatile compounds analysis.

### 2.4. Microbial Analysis

The 5 g samples were mixed with 45 mL of buffered peptone water (Pronadisa, Madrid, Spain) in a filtered bag (Scharlau, Barcelona, Spain) and homogenized using a Pulsifier II (Microgen Bioproducts Ltd., Camberley, Surrey, UK). Decimal dilutions of the filtrate were prepared and spread in triplicate on media plates for microbial counts [11]. Plate count agar (Pronadisa, Madrid, Spain) was used to count total mesophilic bacteria (TMB) and rose bengal agar with chloramphenicol (Scharlau, Barcelona, Spain) to count yeast, both after incubation at 30 °C within 48 h. Results were expressed as log CFU g^−1^ of the model system.

### 2.5. Analysis of Volatile Compounds

The volatile compounds present in the headspace of the sample were analyzed using a gas chromatography/mass spectrometry (GC-MS) 7890–5975 system (Agilent Technologies, Hewlett-Packard, Palo Alto, CA, USA). The device was equipped with an autosampler (MPS2 multipurpose sampler, Gerstel, Mülheim an der Ruhr, Germany) and a DB-624 capillary column (30 m × 0.25 × 1.4 µm, J&W Scientific, Agilent Technologies, USA). The 5 g samples of the supernatant from the models, were introduced into 20 mL headspace vials (Gerstel, Mülheim an der Ruhr, Germany) with a PTFE-faced silicon septum and incubated at 37 °C for 15 min for equilibration (250 rpm speed and 10 s time interval). The compounds were adsorbed onto an 85 µm CAR/PDMS fiber (Supelco, Bellefonte, CA, USA) for 60 min at 37 °C by headspace solid-phase microextraction (HS-SPME) and then, desorbed for 5 min at 240 °C in splitless mode in the injection port of the GC-MS. Helium was used as the carrier gas at a constant flow rate of 0.897 mL/min and a constant average velocity of 34.34 cm/s. The temperature of the GC oven was set at 40 °C for 10 min, then increased to 100 °C at 3 °C/min for 5 min, then increased to 150 °C at 4 °C/min, then to 210 °C at 5 °C/min, and finally to 210 °C for 5 min. The temperature of the MS interface was fixed at 240 °C.

The identification of volatile compounds was performed in full scan mode and by comparing them to the mass spectra from the library database (NIST17). The identity of the compounds was confirmed by comparing their retention time and spectra with those of authentic standard compounds. The retention time index (RI) was calculated using an n-alkane series (C6–C20) under the same analysis conditions [12].

The selected volatile compounds were quantified using standard external calibration curves [13]. Stock standard solutions of pure compounds were prepared in methanol except for those eluting in the initial 5 min, which were prepared in propylene glycol. Then, serial dilutions (1/2, 1/5, 1/10, 1/25, 1/50, 1/100, 1/125, and 1/250) were prepared and analyzed by GC-MS under the same chromatographic conditions. Calibration curves were obtained by plotting the total ion current (TIC) area against the ng of each compound except for 2-methyl butanal, which required the selection of a specific ion (*m*/*z*). Quantification was expressed as ng of the volatile compound extracted by the SPME fiber per g of the meat model. The sensitivity and linearity of the volatile compound analysis were calculated. Standard solutions at six concentration levels, each repeated three times, were used to evaluate the linearity. To determine sensitivity, the limits of detection (LOD) and quantification (LOQ) were calculated from a blank sample (*n* = 5) plus three and ten times the standard deviation, respectively.

The estimation of odor activity values (OAVs) of volatile compounds was calculated from the ratio of the concentration in the meat model by their respective odor threshold reported in the air [14].

### 2.6. Statistical Analysis

The generalized linear model (GLM) method was used to analyze the data using statistical software (XLSTAT 2018, Addinsoft, Barcelona, Spain). The influence of formulations and incubation time was treated as a fixed effect and replicated as a random effect. Tukey’s test was used to compare means when a significant effect (*p* < 0.05) was detected.

## 3. Results and Discussion

**Figure 1:**
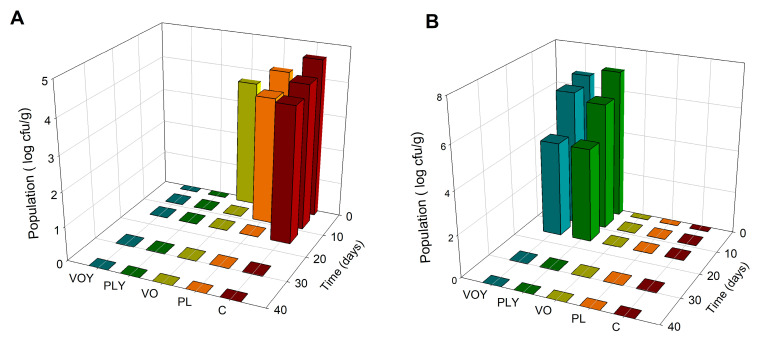
Evolution of the microbial counts (log cfu/g) in the model systems. (**A**) Total mesophilic bacteria and (**B**) yeast. C, Control model system; PL and VO, pork lard and coconut oil model systems, respectively; and PLY and VOY, pork lard and coconut oil model systems inoculated with *D. hansenii*, respectively.

The results of microbial counts in the models are shown in Figure 1. The TMB, present in the C, PL, and VO models, disappeared after 28, 14, and 7 d, respectively. TMB were not detected in the PLY and VOY models, although the inhibitory effect of *D. hansenii* on the growth of TMB had not been observed and requires further research. The absence of yeast after 28 d in the PLY and VOY models indicates the inhibition of yeast growth, probably due to a decrease in the pH of the meat model system at 28 d (Figure S1) because low pH can inhibit the growth of *D. hansenii* [15]. Furthermore, the increase in pH was followed by a sustained decrease in the model, may be due to an increase in alkaline amino acids, followed by an increase in acidic amino acids and fatty acids (Figure S1).

The volatile compounds identified in the five meat model systems are shown in Table S2. Compounds were classified according to their most probable origin, namely carbohydrate fermentation, lipid oxidation, amino acid degradation, esterase activity, lipid β-oxidation, and unknown origins. A total of 63 compounds were identified, and the volatile composition differed among model systems. Two compounds were only present in the C model, 1-octanol, and dimethyl disulfide, whereas 2-methylbutanoic acid was only detected in the PLY model. The VO model was characterized by the presence of three compounds: dodecane, hexadecane, and heptadecane. Furthermore, five compounds were only present in the VOY and PLY models—butanoic acid, 2-methylpropanoic acid, 3-methylbutanoic acid, phenylethyl alcohol, and 2-pentanone.

**Figure 2:**
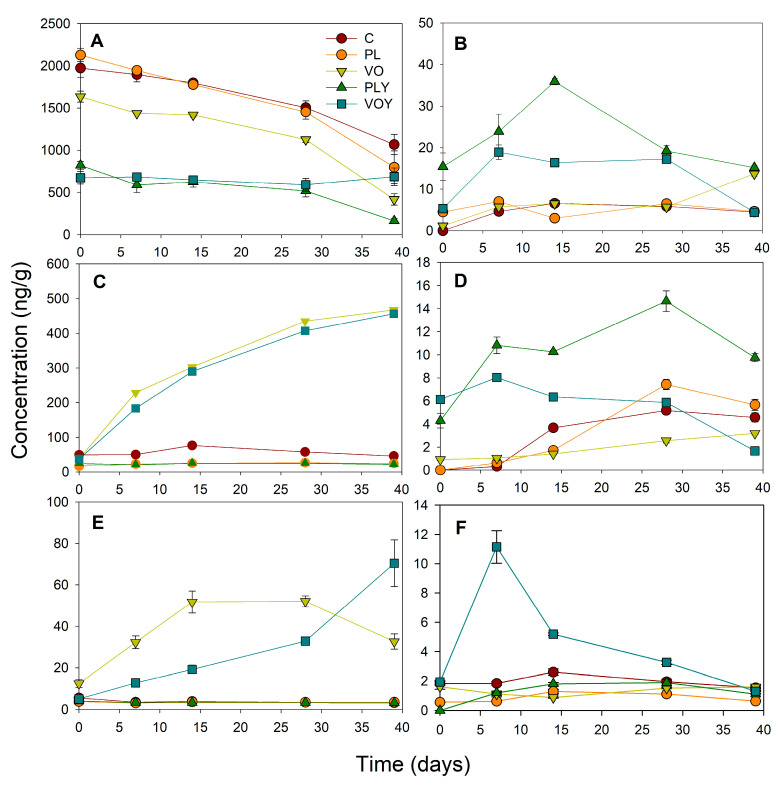
Quantification (ng/g) of volatile compounds in the headspace of model systems classified according to the most likely origin ((**A**), ethanol; (**B**), carbohydrate fermentation (excluding ethanol); (**C**), lipid oxidation; (**D**): amino acid degradation; (**E**), esterase activity; (**F**), lipid β-oxidation reactions). Data are expressed as means ± SE. C, Control model system; PL and VO, pork lard and coconut oil model systems, respectively; and PLY and VOY, pork lard and coconut oil model systems inoculated with *D. hansenii*, respectively.

Considering the odor threshold of the compounds and their importance in the flavor of meat products [16,17], 43 compounds were selected for quantification (Table 1). These volatile compounds were quantified using the external standard method (Table S3). Quantification showed significant differences between the models and along the incubation time (Table S4). The evolution of the content of the volatile compounds grouped according to their probable origin is shown in Figure 2. Ethanol was excluded from the group of compounds derived from carbohydrate degradation due to its considerable abundance compared to the rest of compounds in this group. The ethanol content was significantly higher in the C, PL, and VO models than in the yeast-inoculated models (PLY and VOY) (Figure 2A). This may be related to the highest mesophilic bacteria count in these models. Furthermore, the PLY and VOY models showed higher levels of compounds derived from carbohydrate fermentation (Figure 2B) and amino acid degradation (Figure 2D) during the first 28 d of incubation. In contrast, the models with coconut oil (VO and VOY) showed a higher content of compounds derived from lipid oxidation (Figure 2C) and esterase activity reactions (Figure 2E). The concentration of compounds derived from lipid β-oxidation reactions was only different in the VOY model and at 7 d (Figure 2F).

**Figure 3:**
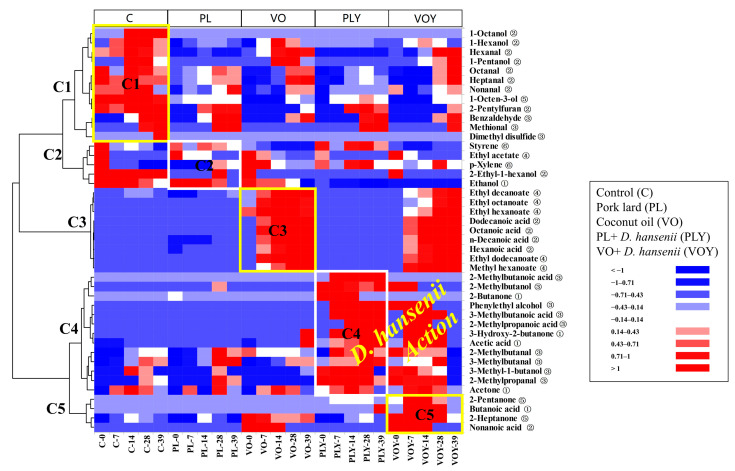
Heatmap with hierarchical clustering based on volatile content in the headspace visualizing differences among model systems. For the map colors, red is used to represent relatively high concentrations, blue represents a relatively low concentration, and white indicates no difference. The numbers (0, 7, 14, 28, and 39) represent the sampling time. C, Control model system; PL and VO, pork lard and coconut oil model systems, respectively; and PLY and VOY, pork lard and coconut oil model systems inoculated with *D. hansenii*, respectively. Compounds were assigned to the most probable origin, as shown in Table S2, and indicated with numbers (①, carbohydrate fermentation; ②, lipid oxidation; ③, amino acid degradation; ④, esterase activity; ⑤, lipid β-oxidation; and ⑥, unknown origin).

The profile of volatile compounds produced in each model system was summarized in a heatmap with hierarchical clustering based on the concentration of volatile compounds quantified in the headspace of the models (Figure 3). Compounds derived from lipid oxidation reactions were clustered in the same group on the top of the heatmap, except for some acid compounds (C1 group, Figure 3). Ester compounds were in the middle of the heatmap (C3 group, Figure 3). Compounds derived from amino acid catabolism (branched-chain aldehydes, alcohols, and acids) were clustered at the bottom of the heatmap (C4 group, Figure 3), together with the compounds derived from carbohydrate degradation reactions. The effect of fat type was associated with a delay in the formation of lipid-derived aldehyde compounds (C1 group, Figure 3) in PL models, while in coconut oil models, the generation of acid compounds and their respective esters (C3 group, Figure 3) increased. Moreover, the effect of yeast activity was associated with the larger production of branched-chain aldehydes and alcohols derived from the amino acid degradation (C4 group, Figure 3). As reported by Bleicher et al. [18], the interaction between products derived from lipid oxidation and Maillard reactions are important for the development of a cooked meat flavour, but it is also essential to study their impact on the aroma of fermented dry-cured systems (Figure 3).

The addition of PL or coconut oil had different effects on the flavor compounds present in the meat model systems. Four acid compounds (hexanoic, octanoic, decanoic, and dodecanoic acids) were detected in the volatile profile of the VO and VOY models, and octanoic acid had the highest content in the models. Hexanoic, octanoic, n-decanoic, and dodecanoic acids have been found in coconut oil in amounts of 0.52%, 7.6%, 5.5%, and 47.7% of total fatty acid methyl esters (FAMEs), respectively [19]. The highest amount of octanoic acid found may be due to the different adsorption kinetics of the SPME fiber [20]. The absence of these compounds in the C, PL, and PLY models is due to the fatty acid composition of the subcutaneous pork lard, which is characterized by mono and unsaturated fatty acids such as palmitic (19.99%), oleic (46.31%), and linoleic (12.96%) acids [21]. A similar trend in the concentration of the four acids (hexanoic, octanoic, n-decanoic, and dodecanoic) was confirmed in the VO and VOY models, as well as their corresponding ethyl esters (Figure 3 and Table S4). The origin of these ethyl esters (ethyl hexanoate and ethyl octanoate) in dry-fermented sausages has been attributed to the inoculation of *D. hansenii* [17]. However, in the PLY model, ethyl hexanoate was not detected, and the content of ethyl octanoate was not significantly different during the incubation time. In contrast, in the VOY model, *D. hansenii* esterase activity increased the amount of these esters’ compounds throughout the incubation time (Table S4); however, an increase in these compounds was detected from days 7 to 28 in the VO model. This may suggest that the most probable origin of these esters compounds in models is a chemical reaction [22].

Yeast inoculation significantly affected the production of compounds derived from carbohydrate fermentation, amino acid degradation, and lipid β-oxidation reactions in both models, although this occurred during the first 14 d of incubation (Figure 2). Compounds derived from carbohydrate degradation reactions were produced by yeast, as observed in the compound 2-butanone in PLY and acetic acid and 3-hydroxy-2-butanone in the PLY and VOY models (Figure 3 and Table S4). This was observed in a model system inoculated with different strains of *D. hansenii* [23]. Furthermore, the abundance of products from the microbial metabolism of valine, leucine, and isoleucine (2-methylpropanal, 3-methylbutanal, and 2-methylbutanal, respectively) [22] were characterized in VOY and PLY up to 28 d. Moreover, the corresponding acids compounds were also present but in lower amounts (Figure 3 and Table S4). These branched acids have been reported in the meat model medium inoculated with *D. hansenii* [24] and a minced meat model [25]. Furthermore, *D. hansenii* also produced notable amounts of phenylethyl alcohol in the VOY and PLY models as a derivative of phenylalanine [26]. In compounds derived from the β-oxidation of fatty acids, only 2-pentanone was produced in PLY and VOY (Figure 3 and Table S4).

The antioxidant effect of *D. hansenii* has been reported in fermented sausages by reducing the contents of heptanal, octanal, and nonanal [5]. In the assayed model systems, the different fats used affected the oxidation process. The yeast in VOY delayed the production of hexanal up to 28 d (Figure 3, Table S4). In the PLY model, heptanal, octanal, and nonanal were reduced by the yeast inoculation, confirming the antioxidant effect of *D. hansenii* yeast; however, no differences in the hexanal content were found.

The different oxidation processes in the models (PL and coconut oil) may have affected the formation of reactive carbonyls derived from lipid oxidation reactions, as well as other reactions with the amino acids present in the models [3,27]. The formation of branched-chain aldehydes is favored by the amino-acid-converting enzymes from yeast [28]. A similar role has been observed in a cheese-surface model inoculated with the yeast strain *D. hansenii* D18335 [29].

Regarding the concentration of amino acids, they were at the same concentration in the models as myofibrillar proteins (Table 1). However, the content of branched-chain aldehydes in the PLY model was higher than in the VOY model (Table S4). This can be explained by the highest content of carbonyl compounds derived from lipid peroxidation, which may have promoted branched-chain aldehyde production [27]. This agrees with the higher content of carbonyl compounds (heptanal, octanal, and nonanal) in the PL and PLY models detected up to 28 d derived from the unsaturated fatty acids present in pork lard [30]. This was also confirmed by the high content of benzaldehyde in PL and PLY (Table S4), which is derived from benzeneacetaldehyde produced from phenylalanine degradation by the action of reactive carbonyls [4]; benzeneacetaldehyde can also be reduced to phenylethyl alcohol [31]. The same effect was observed in methional derived from methionine degradation [10] in the PL and PLY models. Other methionine-derived compounds, such as dimethyl disulfide, were only found in the C model, as already reported in a model containing only free amino acids [1]. The absence of dimethyl disulfide in the VOY and PLY models could be attributed to the antioxidant effect of *D. hansenii* [7], although these compounds may be under the detection limit of our analysis.

**Figure 4:**
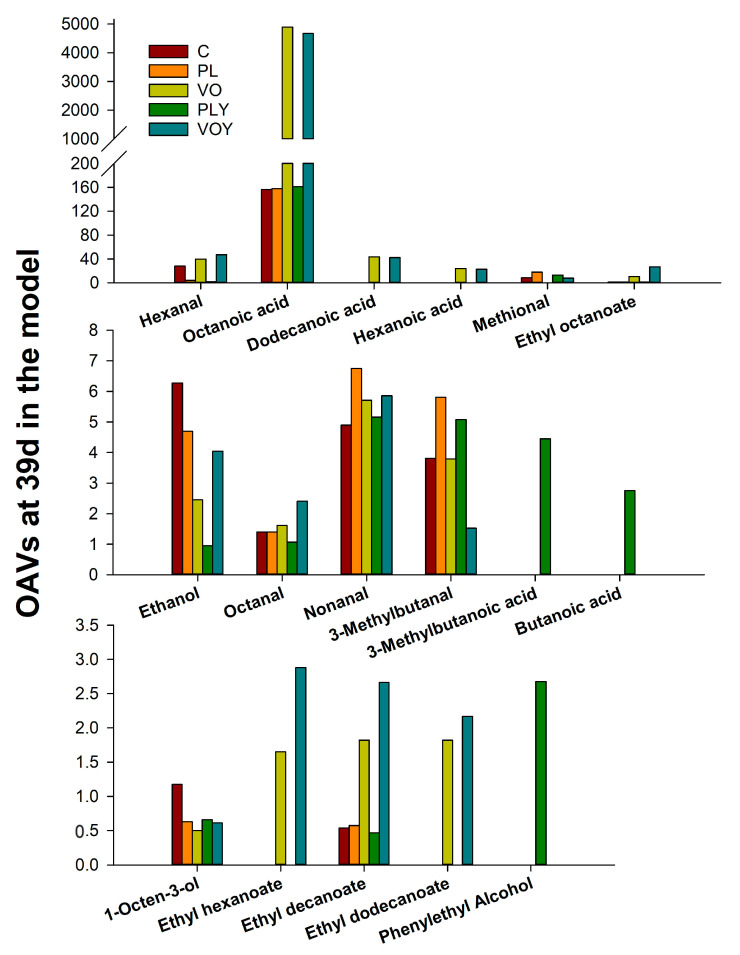
Odor activity values (OAVs > 1) of volatile compounds in the headspace of model systems at 39 d. C, Control model system; PL and VO, pork lard and coconut oil model systems, respectively; PLY and VOY, pork lard and coconut oil model systems inoculated with *D. hansenii*, respectively.

Considering that not all identified compounds may contribute to the aroma of the model, a calculation of their OAV was performed to explain the effect of fat and yeast inoculation on the headspace aroma of models (Table S5). However, it should be considered that the OAVs were calculated based on SPME extraction and using CAR/PDMS fiber; therefore, the OAV profile might change if other extraction techniques were to be used. However, compounds showing OAV > 1 (Figure 4) indicate that they impact the aroma, as their content is above its threshold [32]. Among the compounds quantified, aldehydes (hexanal, octanal, and nonanal) and acids (hexanoic, dodecanoic and octanoic acids) showed the highest OAV indicating the impact of the oxidation reaction on the aroma of the models (Figure 4) [33]. Furthermore, hexanal and the acids octanoic, dodecanoic, and hexanoic had a substantial impact on the aroma of the models containing vegetable oil (VO and VOY). Moreover, their ester compounds, ethyl octanoate, ethyl hexanoate, ethyl decanoate, and ethyl dodecanoate, which contribute to rancid, fatty, green, fruity, and cheesy odors, were also abundant in the VO and VOY models. In contrast, the aroma of models containing PL was affected by octanoic acid (rancid), although additional compounds derived from amino acids also had a high impact, like methional (musty, potato) and 3-methylbutanal (fruity, green, cocoa). Furthermore, yeast inoculation in pork lard models, PLY, increased the presence of phenylethyl alcohol and compounds, such as 3-methylbutanoic and butanoic acids, that contributed to floral and cheesy odor notes, respectively.

## 4. Conclusions

Understanding the reactions involved in the production of key aroma compounds in dry-cured meat products, such as fermented sausages, requires taking into account both the Maillard reactions and lipid oxidation in complex systems, as well as the role of the starters. Both the fat type and yeast strain had a significant effect on the generation of volatile compounds in complex model systems by producing a differential effect on the aroma of the models. The antioxidant and amino-acid-converting effects of *D. hansenii* inoculation were confirmed in both the PLY and VOY models. Furthermore, the different oxidation processes in the models affected the formation of reactive carbonyls derived from lipid oxidation reactions. The highest carbonyl content in the pork lard models (PL and PLY), detected during the initial incubation period (up to 28 d) and derived from unsaturated fatty acids, favored the formation of branched-chain aldehydes in the models. This process was favored by amino-acid-converting enzymes from *D. hansenii*. The production of branched-chain aldehydes, benzaldehyde, and methional in PL models inoculated with *D. hansenii* confirms the action of reactive carbonyls on amino acids. The differences detected among models affected the aroma profile. However, the study of the effect of the oxidation process and yeast metabolism on the promotion of the formation of aromas in complex food systems requires further research due to the many factors involved.

## Figures and Tables

**Table 1:** Physical–chemical parameters and composition (% *w*/*w*) of the model systems.

Meat Model Systems ^1^	C	PL	VO	PLY	VOY
pH	5	5	5	5	5
a_w_^2^	0.895	0.895	0.895	0.895	0.895
NaCl (%)	2.7	2.7	2.7	2.7	2.7
NaNO_3_ (%)	0.0075	0.0075	0.0075	0.0075	0.0075
Glucose (%)	0.5	0.5	0.5	0.5	0.5
Amino acids ^3^	+	+	+	+	+
Myofibrillar proteins (%)	35	35	35	35	35
Pork lard (%)		15		15	
Coconut oil (%)			15		15
Yeast (*D. hansenii* L1) (CFU ml^−1^)				10^6^	10^6^

^1^ C, Control meat model system; PL and VO, pork lard and coconut oil model systems, respectively; PLY and VOY, pork lard and coconut oil model systems inoculated with yeast, respectively. ^2^ The a_w_ was adjusted using glycerol (30 mL/100 g). ^3^ The concentration of amino acids is shown in Table S1.

## Data Availability

Data are contained within the article or Supplementary Materials.

## Supplementary Materials

The following supporting information can be downloaded at: https://www.mdpi.com/article/10.3390/foods12122429/s1, Figure S1: Evolution of pH in model systems. Data expressed as means ± SE; Table S1: The concentration of amino acids (mg/100 mL) in the model systems; Table S2: Volatile compounds detected in the model systems. Table S3: Calibration curves, linearity, and correlation coefficients of volatile compounds quantified in model systems by HS-SPME; Table S4: Quantification (ng/g) of volatile compounds in the headspace of model systems; Table S5: Odor descriptor and OAVs values of volatile compounds in the model systems at 39 days.
