# Supplementary material for: Short-Term Changes in Aroma-Related Volatiles in Meat Model: Effect of Fat and D. hansenii Inoculation
Source: Foods. 2023 Jun 20;12(12):2429. doi: 10.3390/foods12122429 (PMC10297263; doi:10.3390/foods12122429)
Supplement: Supplementary file 1 [file foods-12-02429-s001.zip › foods-2456914-supplementary.pdf]

Article

Short-term changes in aroma-related volatiles in meat model: Effect of fat and *D. hansenii* inoculation

Lei Li, Carmela Belloch, and Mónica Flores *

Figure S1. Evolution of pH in the model systems. Data expressed as means ± SE.

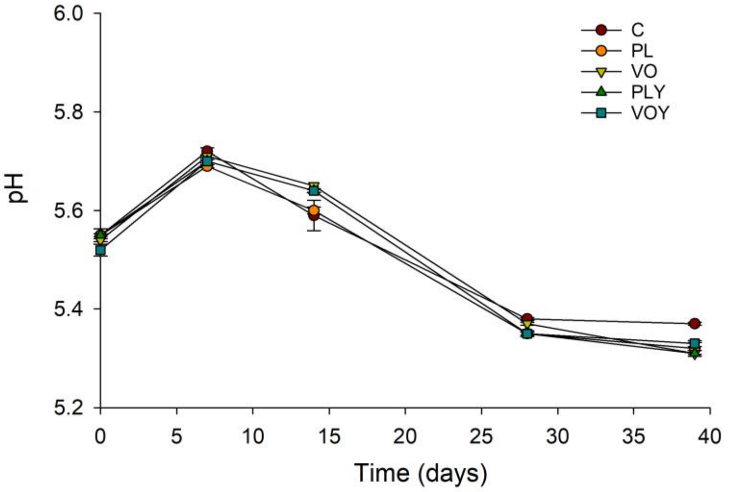

Table S1. The concentration of amino acids (mg/100 mL) in the model systems.

Amino Acids	Concentration (mg/100 mL)	Amino Acids	Concentration (mg/100 mL)	Amino Acids	Concentration (mg/100 mL)	Amino Acids	Concentration (mg/100 mL)
**Ala**	111.8	**Thr**	54.99	**Glu**	73.45	**Tyr**	26.98
**Gly**	42.58	**Ser**	19.24	**Phe**	83.85	**Trp**	17.03
**Val**	90.35	**Pro**	89.05	**Gln**	65	**C-C**	5.36
**β-Ala**	4.54	**Asn**	21.71	**Orn**	31.01	**Arg**	9.49
**Leu**	176.8	**Asp**	100.1	**Lys**	120.9	**Cys**	11.12
**Ile**	62.08	**Met**	36.27	**His**	38.48		

Table S2. Volatile compounds detected in the model systems.

N [1]	Compounds	LRI [2]	LRI [3]	RI [4]	Meat model systems [5]				
					C	PL	VO	PLY	VOY
	Carbohydrate fermentation								
	Acetaldehyde	467	466	a	+ [6]	+	+	+	+
1	Ethanol	508	507	a	+	+	+	+	+
2	Acetone	530	527	a	+	+	+	+	+
3	2-Butanone	635	629	a	-	+	+	+	+
4	Acetic acid	713	714	a	+	+	+	+	+
5	3-Hydroxy-2-butanone	783	783	a	-	-	+	+	+
6	Butanoic acid	888	891	a	-	-	-	+	+
	Lipid oxidation								
	Pentane	500	500	a	+	+	+	+	+
	Hexane	600	600	a	+	+	+	+	+
	Heptane	700	700	a	+	+	+	+	+
	Pentanal	737	736	a	+	+	+	+	+
	2-Methylheptane	767		b	+	+	+	+	+
	4-Methylheptane	768		b	+	+	+	+	+
	3-Methylheptane	775		b	+	+	+	+	+
	Octane	800	800	a	+	+	+	+	+
	2,4-Dimethylheptane	823		b	-	+	-	-	+
7	1-Pentanol	826	823	a	+	-	+	-	+
8	Hexanal	841	839	a	+	+	+	+	+
9	1-Hexanol	924	921	a	+	+	+	+	+
10	Heptanal	942	941	a	+	+	+	+	+
11	2-Pentylfuran	1009	1009	a	+	+	+	+	+
12	Octanal	1048	1044	a	+	+	+	+	+
13	Hexanoic acid	1080	1075	a	+	+	+	+	+
14	2-Ethyl-1-hexanol	1084	1083	a	+	+	+	-	+
15	1-Octanol	1126	1123	a	+	-	-	-	-
16	Nonanal	1151	1148	a	+	+	+	+	+
	Dodecane	1200	1200	a	-	-	+	-	-
17	Nonanoic acid	1360	1350	a	-	-	+	-	+
18	Octanoic acid	1269	1264	a	+	+	+	+	+
19	n-Decanoic acid	1455	1451	a	+	+	+	+	+
	Tetradecane	1399	1400	a	-	-	+	-	+
	Pentadecane	1500	1500	a	-	-	+	-	+
	Hexadecane	1600	1600	a	-	-	+	-	-
20	Dodecanoic acid	1645	1645	a	-	-	+	-	+
	Heptadecane	1700	1700	a	-	-	+	-	-
	Amino acid degradation								

21	2-Methylpropanal	594	590	a	+	+	-	+	+
22	3-Methylbutanal	692	687	a	+	+	+	+	+
23	2-Methylbutanal (58) [7]	701	698	a	+	+	+	+	+
24	Dimethyl disulfide	771	774	a	+	-	-	-	-
25	3-Methyl-1-butanol	795	795	a	+	+	+	+	+
26	2-Methylbutanol	798	795	a	-	+	-	+	+
27	2-Methylpropanoic acid	860	864	a	-	-	-	+	+
28	3-Methylbutanoic acid	939	933	a	-	-	-	+	+
29	2-Methylbutanoic acid	945	947	a	-	-	-	+	-
30	Methional	969	968	a	+	+	-	+	+
31	Benzaldehyde	1018	1013	a	+	+	+	+	+
	Benzeneacetaldehyde	1110	1104	a	+	+	+	+	+
32	Phenylethyl alcohol	1195	1191	a	-	-	-	+	+
	Esterase activity								
33	Ethyl acetate	638	636	a	+	+	+	+	+
34	Methyl hexanoate	953	952	a	-	-	+	-	+
35	Ethyl hexanoate	1028	1026	a	-	-	+	-	+
36	Ethyl octanoate	1229	1226	a	+	+	+	+	+
37	Ethyl decanoate	1427	1421	a	+	+	+	+	+
38	Ethyl dodecanoate	1623	1623	a	-	-	+	-	+
	Lipid β-oxidation								
39	2-Pentanone	733	731	a	-	-	-	+	+
40	2-Heptanone	935	933	a	+	+	+	+	+
	2,3-Octanedione	1028	1029	a	+	-	-	-	+
41	1-Octen-3-ol	1030	1028	a	+	+	+	+	+
	Unknown origin								
	Carbon disulfide	538	532	a	+	+	+	+	+
42	*p*-Xylene	892	892	a	+	+	+	+	+
43	Styrene	920	921	a	+	+	+	+	+
	3-Carene	1021	1026	a	+	+	+	+	+
	D-Limonene	1044	1046	a	+	-	+	+	+

[1] Compounds quantified in the meat model systems.
[2] Linear retention indices (LRI) of the compounds eluted from the GC-MS using a DB-624 capillary column (J & W Scientific 30 m × 0.25 mm i.d. × 1.4 μ m film thickness).
[3] Linear retention indices (LRI) of the authentic standard compounds.
[4] RI: reliability of identification: a, identification by mass spectrum and by coincidence with the LRI of an authentic standard; b, identification by mass spectrum.
[5] C: Control meat model system; PL and VO, pork lard and coconut oil model systems, respectively; PLY and VOY, pork lard and coconut oil model systems inoculated with yeast.
[6] +: detected; -: not detected.
[7] Target ion (m/z) used to quantify the compound when the peak was not completely resolved.

Table S3. Calibration curves, linearity and correlation coefficients of volatile compounds quantified in the model systems by HS-SPME.

Compounds	Measure range (ng)	Slope (A/ng × 10^6)	Intercept (10^6)	Correlation coefficient (r^2)	LOD (ng)	LOQ (ng)
Carbohydrate fermentation						
Ethanol	304.40-15220	0.04	28.49	0.9967	0.00	0.00
Acetone	2.96-74	0.10	-0.02	0.9991	0.00	0.00
2-Butanone	0.78-195	0.26	0.60	0.9998	0.00	0.00
Acetic acid	3.90-78	0.43	-1.97	0.9879	5.87	7.16
3-Hydroxy-2-butanone	9.60-240	0.16	-2.55	0.9968	11.72	12.75
Butanoic acid	6.32-31.60	0.38	-1.41	0.9981	4.98	7.03
Lipid oxidation						
1-Pentanol	1.18-11.8	0.61	-0.20	0.996	0.00	0.00
Hexanal	1.88-470	0.39	0.12	0.9994	1.23	2.90
1-Hexanol	1.32-16.5	1.13	-1.19	0.9967	1.56	2.35
Heptanal	0.8-20	0.74	-0.56	0.9991	1.07	1.53
2-Pentylfuran	2.3-57.5	0.21	-0.80	0.9915	0.00	0.00
Octanal	1.72-17.2	0.22	-0.32	0.9941	0.00	0.00
Hexanoic acid	10.24-512	0.65	-9.98	0.9968	16.09	16.39
2-Ethyl-1-hexanol	1.32-6.6	0.87	-0.78	0.9951	0.00	0.00
1-Octanol	4.1-41	1.47	-5.60	0.9954	0.00	0.00
Nonanal	4.3-43	0.81	-2.05	0.9994	2.81	3.14
Nonanoic acid	2.64-13.2	1.36	-3.69	0.982	0.00	0.00
Octanoic acid	34.36-1718	0.53	-25.60	0.9971	0.00	0.00
n-Decanoic acid	9.84-123	0.78	-7.19	0.9984	0.00	0.00
Dodecanoic acid	21.6-216	0.51	-14.98	0.982	32.13	34.41
Amino acid degradation						
2-Methylpropanal	1.56-39	0.13	-0.14	0.9933	2.45	3.97
3-Methylbutanal	0.88-22	0.41	0.25	0.9992	0.00	0.00

2-Methylbutanal (58)[a]	0.84-21	0.04	-0.01	0.9997	0.00	0.00
Dimethyl disulfide	1.56-15.6	0.10	-0.04	0.9958	1.71	2.59
3-Methyl-1-butanol	1.84-46	0.54	-1.11	0.9942	2.42	2.81
2-Methylbutanol	1.11-11.41	1.06	-0.05	0.9989	0.20	0.32
2-Methylpropanoic acid	6.48-32	0.33	-0.89	0.9891	4.05	5.76
3-Methylbutanoic acid	2.69-13.4	0.41	-1.07	0.9868	0.00	0.00
2-Methylbutanoic acid	5.32-26.6	0.51	-2.37	0.9924	5.88	7.89
Methional	2.04-25.5	0.37	-0.75	0.9917	2.41	2.76
Benzaldehyde	0.94-9.4	0.82	-0.74	0.9053	1.97	3.86
Phenylethyl alcohol	2.65-26.5	0.80	-2.97	0.9892	4.06	4.43
Esterase activity						
Ethyl acetate	1.9-19	0.37	0.02	0.997	0.00	0.00
Methyl hexanoate	2.08-10.4	0.26	-0.16	0.9929	0.00	0.00
Ethyl hexanoate	4-100	0.36	-1.90	0.9948	0.00	0.00
Ethyl octanoate	6.64-415	0.44	-5.10	0.9985	12.50	13.21
Ethyl decanoate	2.4-48	0.70	-1.84	0.9968	0.00	0.00
Ethyl dodecanoate	5.3-106	0.24	-1.44	0.9976	0.00	0.00
Lipid β-oxidation						
2-Pentanone	1.32-66	0.48	-0.24	0.9978	0.00	0.00
2-Heptanone	1.2-30	0.49	-0.68	0.9986	2.36	3.78
1-Octen-3-ol	2.48-31	0.85	-1.84	0.9965	0.00	0.00
Unknown origin						
p-Xylene	0.82-8.2	0.32	-0.04	0.9884	0.00	0.00
Styrene	0.52-5.2	0.58	-0.29	0.9938	0.87	1.35

[a] Target ion (m/z) used to quantify the compound.

Table S4. Quantification (ng/g) of volatile compounds in the headspace of model systems.

Group	Times (days)	Ethanol		Acetone		2-Butanone		Acetic acid		3-Hydroxy-2-butanone		Butanoic acid		*Carbohydrate fermentation (excluding ethanol)*	
C [a]	0	1972.84	ab [d]	- [e]		-		-		-		-		-	
	7	1896.25	abc	4.64	ab	-		-		-		-		4.64	de
	14	1797.97	abcd	5.44	a	-		1.13	fg	-		-		6.57	d
	28	1504.81	cde	4.68	ab	-		1.17	fg	-		-		5.85	de
	39	1067.24	fgh	3.35	de	-		1.15	fg	-		-		4.50	de
PL	0	2127.34	a	-		2.28	c	1.16	fg	-		-		3.44	de
	7	1944.64	ab	4.78	ab	1.08	c	1.17	fg	-		-		7.02	d
	14	1777.99	abcd	1.30	f	0.59	c	1.13	fg	-		-		3.02	de
	28	1452.55	def	5.30	ab	-		1.18	fg	-		-		6.49	de
	39	798.75	ghi	3.29	de	-		1.37	fg	-		-		4.66	de
VO	0	1635.58	bcd	-		1.17	c	-		-		-		1.17	de
	7	1436.30	def	4.26	bcd	0.30	c	1.18	fg	-		-		5.75	de
	14	1417.96	def	5.23	ab	0.13	c	1.14	fg	-		-		6.51	d
	28	1125.76	efg	4.50	abc	-		1.22	fg	-		-		5.71	de
	39	416.63	ij	2.82	e	-		6.58	abcd	4.32	c	-		13.72	c
PLY	0	820.99	ghi	3.57	cde	10.27	b	1.58	fg	-		-		15.42	c
	7	589.19	i	4.51	abc	11.79	b	3.16	ef	4.36	bc	-		23.83	b
	14	625.11	i	5.47	a	21.26	a	4.39	de	4.78	abc	-		35.89	a
	28	517.15	ij	4.56	abc	0.49	c	9.00	a	5.15	ab	-		19.20	bc
	39	161.43	j	2.88	e	-		6.05	bcd	5.28	a	0.96	a	15.17	c
VOY	0	673.61	hi	3.03	e	0.71	c	1.57	fg	-		-		5.31	de
	7	683.71	hi	4.99	ab	0.29	c	7.88	ab	4.69	abc	1.05	a	18.90	bc
	14	647.55	i	5.47	a	0.09	c	5.25	cde	4.67	abc	0.93	a	16.41	c
	28	590.91	i	4.84	ab	-		7.12	abc	4.25	c	1.02	a	17.22	c
	39	687.08	hi	2.95	e	-		1.46	fg	-		-		4.41	de
	**RMSE** [b]	132.35		0.33		1.88		0.78		0.26		0.05		2.05	
	***P*g** [c]	***		***		***		***		***		***		***	
	***P*t** [c]	***		***		***		***		***		***		***	
	***P*g*t** [c]	***		***		***		***		***		***		***	

Group	Times (days)	1-Pentanol		Hexanal		1-Hexanol		Heptanal		2-Pentylfuran		Octanal		Hexanoic acid	
C [a]	0	-		24.75	def	0.28	de	0.87	abc	1.75	bcd	1.72	ab	3.57	d
	7	-		28.45	cde	0.65	bcd	0.72	bcdef	1.47	bcd	1.06	cdefghi	3.43	d
	14	1.54	a	46.68	ab	1.61	a	1.19	a	2.62	a	2.17	a	3.43	d
	28	1.62	a	31.70	cd	1.03	bc	0.72	bcdef	2.25	ab	1.53	bc	3.50	d
	39	0.37	cd	23.06	defg	0.56	cd	0.74	bcdef	2.21	ab	1.23	bcdefg	3.42	d
PL	0	-		4.05	hi	-		0.55	bcdef	-		0.86	defghi	-	
	7	-		6.16	hi	0.30	de	0.56	bcdef	-		1.03	cdefghi	3.31	d
	14	-		7.06	hi	0.39	de	0.66	bcdef	1.46	bcd	1.17	bcdefgh	3.32	d
	28	-		5.54	hi	0.40	de	0.68	bcdef	1.62	bcd	1.33	bcde	3.41	d
	39	-		3.36	i	0.25	de	0.67	bcdef	1.61	bcd	1.23	bcdefg	3.45	d
VO	0	-		9.66	ghi	-		0.42	ef	-		0.82	efghi	4.78	d
	7	-		16.95	efgh	0.44	de	0.48	cdef	-		0.79	efghi	24.88	c
	14	0.89	b	34.36	bcd	1.07	b	0.51	bcdef	-		0.88	defghi	36.97	b
	28	1.42	a	57.78	a	0.56	cd	0.79	abcde	-		1.40	bcd	60.89	a
	39	0.15	de	32.53	cd	0.34	de	0.84	abcd	0.98	d	1.42	bcd	69.75	b
PLY	0	-		2.63	i	0.34	de	0.47	cdef	-		0.65	hi	3.59	d
	7	-		1.83	i	0.40	de	0.41	ef	-		0.76	fghi	3.35	d
	14	-		3.97	hi	0.40	de	0.57	bcdef	1.73	bcd	0.96	defghi	3.37	d
	28	-		3.50	i	0.36	de	0.62	bcdef	1.97	abc	1.10	cdefghi	3.50	d
	39	-		1.72	i	0.28	de	0.60	bcdef	1.55	bcd	0.94	defghi	3.46	d
VOY	0	-		8.18	hi	0.31	de	0.43	ef	-		0.78	efghi	4.25	d
	7	-		5.25	hi	0.50	de	0.33	f	-		0.58	i	23.02	c
	14	0.24	de	12.35	fghi	0.52	d	0.44	def	-		0.71	ghi	37.31	b
	28	0.51	c	39.04	bc	0.42	de	0.71	bcdef	-		1.31	bcdef	63.58	a
	39	0.90	b	38.75	bc	0.27	de	0.89	ab	1.25	cd	2.12	a	66.43	a
	RMSE [b]	0.08		4.24		0.16		0.13		0.26		0.18		3.28	
	*P*g [c]	***		***		***		***		***		***		***	
	*P*t [c]	***		***		***		***		***		***		***	
	*P*g*t [c]	***		***		***		***		***		***		***	

Group	Times (days)	2-Ethyl-1-hexanol		1-Octanol		Nonanal		Nonanoic acid		Octanoic acid		n-Decanoic acid		Dodecanoic acid		*Lipid oxidation*	
C [a]	0	0.57	a	-		1.92	bcd	-		12.27	d	2.13	f	-		49.83	d
	7	0.39	ab	-		1.89	bcd	-		10.72	d	1.94	f	-		50.72	d
	14	0.48	ab	0.98	c	3.24	a	-		10.87	d	1.97	f	-		76.79	d
	28	0.41	ab	0.92	b	2.32	b	-		10.37	d	1.96	f	-		58.35	d
	39	0.38	ab	0.91	b	1.47	cde	-		10.18	d	1.95	f	-		46.47	d
PL	0	-		-		1.31	cde	-		9.97	d	-		-		16.74	d
	7	-		-		1.57	bcde	-		10.04	d	-		-		22.97	d
	14	-		-		1.43	cde	-		10.07	d	-		-		25.57	d
	28	0.42	ab	-		1.70	bcde	-		10.09	d	1.90	f	-		27.09	d
	39	-		-		2.02	bc	-		10.25	d	1.97	f	-		24.82	d
VO	0	0.35	b	-		1.29	cde	0.60	d	18.84	d	2.06	f	-		38.80	d
	7	-		-		1.34	cde	0.64	abc	163.55	bc	11.70	de	8.38	d	229.15	bc
	14	-		-		1.31	cde	0.64	abc	198.24	b	15.90	cd	11.78	c	302.55	d
	28	-		-		1.70	bcde	-		277.00	a	19.21	bc	14.18	bc	434.92	a
	39	-		-		1.71	bcde	-		318.05	a	24.39	a	17.43	a	467.62	a
PLY	0	-		-		0.95	e	-		13.94	d	2.15	f	-		24.71	d
	7	-		-		1.51	bcde	-		11.22	d	1.94	f	-		21.43	d
	14	-		-		1.53	bcde	-		11.06	d	1.95	f	-		25.54	d
	28	-		-		1.45	cde	-		10.90	d	2.17	f	-		25.59	d
	39	-		-		1.55	bcde	-		10.50	d	1.95	f	-		22.56	d
VOY	0	0.34	b	-		1.74	bcde	0.63	bc	19.04	d	2.15	f	-		37.84	d
	7	-		-		1.15	de	0.67	d	134.38	c	9.29	e	8.18	d	183.35	c
	14	-		-		1.42	cde	0.67	ab	208.18	d	15.97	cd	12.23	c	290.03	bc
	28	-		-		1.58	bcde	-		265.60	a	19.90	abc	14.43	bc	407.09	a
	39	-		-		1.76	bcd	-		303.38	a	23.68	ab	16.95	ab	456.37	a
	RMSE [b]	0.06		0.02		0.25		0.01		17.61		1.47		0.88		23.70	
	***P*g** [c]	***		***		***		***		***		***		***		***	
	***P*t** [c]	***		***		***		***		***		***		***		***	
	***P*g*t** [c]	***		***		***		***		***		***		***		***	

Group	Times (days)	2-Methylpropanal		3-Methylbutanal		2-Methylbutanal		Dimethyl disulfide		3-Methyl-1-butanol		2-Methylbutanol		2-Methylpropanoic acid	
C [a]	0	-		-		-		-		-		-		-	
	7	-		0.32	kl	-		-		-		-		-	
	14	-		0.80	hijk	0.59	efg	-		1.96	cde	-		-	
	28	0.63	de	1.51	cdef	0.73	bcdef	-		1.11	fg	-		-	
	39	0.47	ef	1.33	defghi	0.63	efg	0.47	a	-		-		-	
PL	0	-		-		-		-		-		-		-	
	7	-		0.37	kl	0.27	fg	-		-		-		-	
	14	-		0.90	fghijk	0.53	efg	-		-		-		-	
	28	1.00	bcd	2.27	b	1.37	abc	-		1.28	efg	0.15	d	-	
	39	0.71	cde	2.03	bc	0.87	bcdef	-		-		-		-	
VO	0	-		-		0.94	bcde	-		-		-		-	
	7	-		0.42	jkl	0.63	efg	-		-		-		-	
	14	-		0.75	ijk	0.67	def	-		-		-		-	
	28	-		1.44	cdefg	0.72	cdef	-		-		-		-	
	39	-		1.33	defghi	0.63	efg	-		0.69	gh	-		-	
PLY	0	1.17	abc	0.88	ghijk	-		-		1.92	cde	0.31	b	-	
	7	1.20	ab	1.31	defghi	1.38	ab	-		3.59	a	0.49	a	-	
	14	1.14	abc	1.40	defghi	0.94	bcde	-		2.88	ab	0.24	bcd	0.87	bc
	28	1.59	a	3.38	a	1.71	a	-		2.61	bc	0.18	cd	1.01	ab
	39	0.63	de	1.78	bcde	0.81	bcdef	-		-		0.49	a	1.08	a
VOY	0	1.12	abc	0.79	hijk	1.30	abcd	-		1.79	def	0.29	bc	-	
	7	1.05	bcd	1.18	efghi	0.96	bcde	-		2.24	bcd	0.27	bcd	0.81	cd
	14	1.02	bcd	1.03	fghij	0.87	bcdef	-		1.28	efg	-		0.68	d
	28	1.05	bcd	1.91	bcd	0.89	bcdef	-		1.03	fg	-		-	
	39	-		0.53	jkl	-		-		-		-		-	
	RMSE [b]	0.15		0.20		0.21		0.03		0.25		0.04		0.06	
	Pg [c]	***		***		***		***		***		***		***	
	Pt [c]	***		***		***		***		***		***		***	
	*Pg**t [c]	***		***		***		***		***		***		***	

Group	Times (days)	3-Methylbutanoic acid		2-Methylbutanoic acid		Methional		Benzaldehyde		Phenylethyl Alcohol		*Amino acid degradation*	
**C** [a]	0	-		-		-		-		-		-	
	7	-		-		-		-		-		0.32	m
	14	-		-		-		0.32	e	-		3.67	hij
	28	-		-		0.48	e	0.72	bc	-		5.17	fgh
	39	-		-		0.56	de	1.11	a	-		4.57	fghi
**PL**	0	-		-		-		-		-		0.00	m
	7	-		-		-		-		-		0.64	m
	14	-		-		-		0.31	e	-		1.73	klm
	28	-		-		0.70	bc	0.69	bc	-		7.45	de
	39	-		-		1.13	a	0.91	ab	-		5.65	efg
**VO**	0	-		-		-		-		-		0.94	lm
	7	-		-		-		-		-		1.05	lm
	14	-		-		-		-		-		1.42	klm
	28	-		-		-		0.41	de	-		2.57	jkl
	39	-		-		-		0.55	cde	-		3.19	ijk
**PLY**	0	-		-		-		-		-		4.29	ghij
	7	0.77	ab	1.14	b	-		-		0.94	a	10.82	b
	14	0.75	ab	1.16	ab	-		-		0.87	bc	10.25	b
	28	0.83	ab	1.20	ab	0.64	cd	0.61	cd	0.90	ab	14.65	a
	39	0.98	a	1.49	a	0.83	b	0.75	bc	0.94	d	9.76	bc
**VOY**	0	-		-		-		-		0.83	c	6.13	ef
	7	0.62	b	-		-		-		0.91	ab	8.04	cd
	14	0.61	b	-		-		-		0.87	bc	6.35	def
	28	0.58	b	-		-		0.40	de	-		5.87	efg
	39	-		-		0.50	e	0.64	cd	-		1.67	klm
**RMSE** [b]		**0.08**		**0.11**		**0.04**		**0.08**		**0.02**		**0.58**	
***P*g** [c]		***		***		***		***		***		***	
***P*t** [c]		***		***		***		***		***		***	
***P*g*t** [c]		***		***		***		***		***		***	

Group	Times (days)	Ethyl Acetate		Methyl hexanoate		Ethyl hexanoate		Ethyl octanoate		Ethyl decanoate		Ethyl dodecanoate		*Esterase activity*	
C [a]	0	1.93	a	-		-		2.96	e	0.63	g	-		5.52	de
	7	-		-		-		2.78	e	0.64	g	-		3.43	de
	14	-		-		-		2.92	e	0.87	fg	-		3.79	de
	28	-		-		-		2.68	e	0.72	g	-		3.40	e
	39	-		-		-		2.53	e	0.65	g	-		3.18	e
PL	0	1.32	abc	-		-		2.44	e	-		-		3.76	de
	7	0.39	de	-		-		2.64	e	-		-		3.03	e
	14	0.27	de	-		-		2.72	e	0.60	g	-		3.59	de
	28	-		-		-		2.79	e	0.64	g	-		3.43	e
	39	-		-		-		2.77	e	0.69	g	-		3.45	e
VO	0	1.65	ab	-		3.79	cde	6.11	e	0.84	fg	-		12.39	de
	7	0.46	cde	0.18	bc	7.44	ab	20.47	cd	1.35	de	2.37	cd	32.27	c
	14	0.21	de	0.27	bc	7.93	ab	37.85	b	2.09	bc	3.38	b	51.73	b
	28	-		0.50	ab	7.96	a	36.70	b	2.47	g	4.41	a	52.04	b
	39	-		0.61	ab	4.95	cd	21.21	c	2.18	g	3.64	b	32.59	c
PLY	0	0.54	cde	-		-		2.87	e	0.58	g	-		3.99	de
	7	-		-		-		2.68	e	0.57	g	-		3.25	e
	14	-		-		-		2.64	e	0.57	g	-		3.21	e
	28	-		-		-		2.58	e	0.64	g	-		3.21	e
	39	-		-		-		2.55	e	0.56	g	-		3.11	e
VOY	0	1.01	bcd	-		-		3.37	e	0.66	g	-		5.04	de
	7	0.39	de	0.37	abc	1.98	ef	7.10	de	0.97	efg	1.92	d	12.73	de
	14	-		0.51	ab	2.84	de	12.16	cde	1.29	def	2.43	de	19.23	cd
	28	-		0.52	ab	5.50	bc	22.42	c	1.68	cd	2.70	c	32.83	c
	39	-		0.78	a	8.64	a	53.48	a	3.19	a	4.34	a	70.44	a
	RMSE [b]	0.29		0.14		0.78		4.34		0.14		0.20		4.96	
	Pg [c]	*		***		***		***		***		***		***	
	Pt [c]	***		***		***		***		***		***		***	
	*Pg**t [c]	***		***		***		***		***		***		***	

Group	Times (days)	2-Pentanone		2-Heptanone		1-Octen-3-ol		*Lipid β-oxidation*		*p*-Xylene		Styrene		*Unknown*	
**C** [a]	0	-		-		1.83	a	1.83	def	0.45	ab	1.57	a	2.02	a
	7	-		0.45	efg	1.39	b	1.84	def	0.14	fgh	0.29	bcdef	0.43	efg
	14	-		0.71	def	1.90	a	2.61	cd	0.13	gh	0.41	bcdef	0.54	cdefg
	28	-		0.52	ef	1.43	b	1.95	cde	-		0.29	cdef	0.29	fg
	39	-		0.37	fg	1.17	b	1.54	def	-		0.15	f	0.15	g
**PL**	0	-		-		0.56	c	0.56	fg	-		0.52	bcdef	0.52	defg
	7	-		-		0.63	c	0.63	efg	-		0.33	bcdef	0.33	fg
	14	-		0.64	def	0.65	c	1.30	defg	-		0.57	bcdef	0.57	cdefg
	28	-		0.44	efg	0.68	c	1.12	efg	0.26	cdefg	0.44	bcdef	0.70	bcdef
	39	-		-		0.63	c	0.63	efg	0.20	efg	0.36	bcdef	0.56	cdefg
**VO**	0	-		1.61	ab	-		1.61	def	0.54	a	0.32	bcdef	0.86	bcde
	7	-		1.12	bcd	-		1.12	efg	0.39	abc	0.22	def	0.61	bcdef
	14	-		0.88	de	-		0.88	efg	0.19	efg	0.14	f	0.34	fg
	28	-		0.91	de	0.61	c	1.52	def	0.15	fgh	0.14	f	0.30	fg
	39	-		1.10	cd	0.50	c	1.60	def	0.15	fgh	0.13	f	0.28	fg
**PLY**	0	-		-		-		-		0.31	bcdef	0.67	bc	0.98	bc
	7	0.52	cd	-		0.67	c	1.19	efg	0.16	fgh	0.27	def	0.43	efg
	14	0.55	cd	0.57	ef	0.69	c	1.81	def	0.33	bcde	0.62	bcd	0.95	bcd
	28	0.42	cd	0.74	def	0.73	c	1.90	def	0.37	bcd	0.69	b	1.06	b
	39	-		0.45	efg	0.66	c	1.11	efg	0.22	defg	0.43	bcdef	0.65	bcdef
**VOY**	0	0.43	cd	1.50	abc	-		1.94	cde	0.20	efg	0.17	ef	0.37	fg
	7	9.23	a	1.91	a	-		11.15	a	0.15	fgh	0.15	f	0.30	fg
	14	3.69	b	1.50	abc	-		5.19	b	0.19	efg	0.14	f	0.33	fg
	28	1.18	c	1.48	abc	0.61	c	3.27	c	0.35	bcde	0.18	ef	0.53	defg
	39	-		0.69	def	0.61	c	1.30	defg	0.20	efg	0.16	f	0.36	fg
	**RMSE** [b]	0.30		0.16		0.09		0.43		0.05		0.12		0.14	
	Pg [c]	***		***		***		***		***		***		***	
	Pt [c]	***		***		***		***		***		***		***	
	Pg*t [c]	***		***		***		***		***		***		***	

[a] C: Control meat model system; PL and VO: pork lard and coconut oil meat model systems, respectively; PLY and VOY: pork lard and coconut oil meat model systems with inoculated yeast.

[b] RMSE: root mean square error.

[c] *P* value of group (g), ripening time (t) and group and ripening time (g*t) effect at ***: $P < 0.001$, **: $P < 0.01$, *: $P < 0.05$, ns: $P > 0.05$.

[d] Different letters in columns indicate significant differences at $P < 0.05$.

[e] -: not detected

Table S5. Odor descriptor and OAVs values of volatile compounds in the model systems at 39 days.

Compounds	Odor descriptor [a]	Threshold in air (ng/g) [b]	Meat model systems [d]				
			C	PL	VO	PLY	VOY
Carbohydrate fermentation							
Ethanol	Alcoholic	170–76000000	6.3	4.7	2.5	<1	4.0
Acetone	Solvent	1100-27900000	<1	<1	<1	<1	<1
2-Butanone	Fruity, green	750-1000000	<1	<1	<1	<1	<1
Acetic acid	Pungent, sour, fruit	10-500000	<1	<1	<1	<1	<1
3-Hydroxy-2-butanone	Creamy, dairy, sweet	14-10000[c]	<1	<1	<1	<1	<1
Butanoic acid	Acidic, sour, cheesy	0.35-9000	<1	<1	<1	2.8	<1
Lipid oxidation							
1-Pentanol	Pungent, fermented, bready	20-1100000	<1	<1	<1	<1	<1
Hexanal	Green, woody, vegetable	0.82-84500	28.1	4.1	39.7	2.1	47.3
1-Hexanol	Green, fruity, apple	10-65000	<1	<1	<1	<1	<1
Heptanal	Green, aldehydic, oily	0.85-9500	<1	<1	1.0	<1	1.0
2-Pentylfuran	Green, waxy, musty	19-270	<1	<1	<1	<1	<1
Octanal	Aldehydic, green, peely	0.88-5000	1.4	1.4	1.6	1.1	2.4
Hexanoic acid	Cheesy, fruity, phenolic	2.9-3500	1.2	1.2	24.1	1.2	22.9
2-Ethyl-1-hexanol	Sweet, fatty, fruity	198-25482.2	<1	<1	<1	<1	<1
1-Octanol	Green, citrus, orange	5-9000	<1	<1	<1	<1	<1
Nonanal	Aldehydic, citrus, cucumber	0.3-230	4.9	6.7	5.7	5.2	5.9
Nonanoic acid	Cheesy, sweet, creamy	1.6-120	<1	<1	<1	<1	<1
Octanoic acid	Rancid, soapy, cheesy	0.065-3200	156.6	157.7	4893.2	161.5	4667.4
n-Decanoic acid	Soapy, waxy, fruity	50-90	<1	<1	<1	<1	<1
Dodecanoic acid	Fatty, coconut, bay	0.4-100	<1	<1	43.6	<1	42.4
Amino acid degradation							
2-Methylpropanal	Fresh, aldehydic, floral, pungent	1-410	<1	<1	<1	<1	<1

3-Methylbutanal	Fruity, dry, green, cocoa	0.35-6	3.8	5.8	3.8	5.1	1.5
2-Methylbutanal	Musty, rummy, nutty	100	<1	<1	<1	<1	<1
Dimethyl disulfide	Sulfurous, cabbage, malty, creamy	1.1-5600	<1	<1	<1	<1	<1
3-Methyl-1-butanol	Fusel, fermented, fruity	19–6300	<1	<1	<1	<1	<1
2-Methylbutanol	Ethereal fresh	140-329000	<1	<1	<1	<1	<1
2-Methylpropanoic acid	Acidic, sour, cheesy	5-240	<1	<1	<1	<1	<1
3-Methylbutanoic acid	Cheesy, dairy, acidic, sour	0.22-3000	<1	<1	<1	4.4	<1
2-Methylbutanoic acid	Fruity, dirty, acidic	20	<1	<1	<1	<1	<1
Methional	Musty, tomato, potato	0.063-60	8.9	18.0	<1	13.2	7.9
Benzaldehyde	Sharp, sweet, bitter	10-3400000	<1	<1	<1	<1	<1
Phenylethyl alcohol	Sweet, floral, fresh	0.35-3800	<1	<1	<1	2.7	<1
Esterase activity							
Ethyl acetate	Ethereal, fruity, sweet	340-1120000	<1	<1	<1	<1	<1
Methyl hexanoate	Ethereal, fruity, pineapple	39-87 [c]	<1	<1	<1	<1	<1
Ethyl hexanoate	Sweet, fruity, pineapple	3-18100	<1	<1	1.7	<1	2.9
Ethyl octanoate	Fruity, winey, waxy, sweet	2-40	1.3	1.4	10.6	1.3	26.7
Ethyl decanoate	Sweet, waxy, fruity, apple	1.2-530	<1	<1	1.8	<1	2.7
Ethyl dodecanoate	Sweet, waxy, floral, soapy	2	<1	<1	1.8	<1	2.2
Lipid β-oxidation							
2-Pentanone	Sweet, fruity, ethereal, winey	98-230000	<1	<1	<1	<1	<1
2-Heptanone	Cheesy, fruity, ketonic, green	3.5-330	<1	<1	<1	<1	<1
1-Octen-3-ol	Mushroom, earthy, green	1-110	1.2	<1	<1	<1	<1
Unknown origin							
p-Xylene		250-9100	<1	<1	<1	<1	<1
Styrene	Sweet, balsamic, floral	12-258000	<1	<1	<1	<1	<1

[a] Odor descriptors from The Good Scents Company database (www.thegoodscentscompany.com).
[b] Threshold values in air obtained from Van Gemert (2011).
[c] Threshold values in water obtained from Van Gemert (2011).

[d] C: Control meat model system; PL and VO: pork lard and coconut oil meat model systems, respectively; PLY and VOY: pork lard and coconut oil meat model systems with inoculated yeast.
